# Professor Unna's Gelatines

**Published:** 1894-02-10

**Authors:** 


### New Drugs and Preparations.

### PROFESSOR UNNA'S GELATINES.

This is a method of dressing eczema, devised some years ago by Professor Unna, of Hamburg. During the last three years we have used them more frequently than any other form of dressing, and with increasing satisfaction to ourselves and the patient.

The " gelatines " consist of a base of gelatine combined with oxide of zinc, to which other drugs may be added. The gelatine is manufactured in small cubes, which are melted in a water bath (such as an ordinary glue pot). It readily becomes of the consistency of cream, and is then painted on the surface with a soft brush; not too large, as that carries so much hot material; an ordinary throat brush does well. Immediately on application it begins to set into a thin elastic skin, quite hard enough to protect the surface. Before it is quite dry it should be dabbed over with a loose pad of absorbent cotton wool, which forms an outer coat like lint. Some discretion must be used not to apply the preparation too hot. No cotton wool need be applied on the face. After a day or two, according to the friction to which the coating is exposed, it must be renewed; any worn patches can be painted over without disturbing the old coating. The gelatine is readily washed off with hot water, or may be picked off when the skin beneath is repaired.

The gelatine coat is sufficiently porous to allow discharge to come through, and it then hardens on the outside, not in contact with the inflamed dermis.

These gelatines are prepared by Messrs. Bell, 225, Oxford Street. The forms we have used most, which are kept in stock by them, contain oxide of zinc alone (" Gelatinum Zinci "), or combined with two and a-half per cent. of ichthyol, or two and a-half per cent. of extract of Cannibis Indica, or with both of these drugs. These gelatines we have found to keep over a year in tin boxes, but Mr. Bell informs us they are rather liable to mould. These preparations are particularly useful in acute eczema of the face, affording a means of applying the remedies far superior to a mask of lint soaked in any lotion. The elastic quality of this gelatine skin is of great value mechanically, as by its gentle uniform pressure in drying it lessens the hyperæmia of the skin which is present in acute eczema.

Another very useful preparation of Professor Unna's is his "Plaster Mull," No. 88; also kept by Mr. Bell. It consists of a strong plaster spread on gauze, containing mercury, perchloride of mercury, carbolic acid, and oxide of zinc. It forms an admirable antiseptic strapping for small incised wounds, or for the later dressing of operation wounds. The writer of this note always carries it in his emergency bag, and has constantly used it for some years for these purposes. It is also a most useful application for cutting short the formation of boils in an early stage; the whole area of the boil must be covered with it, and no hole cut as in the usual protective plaster.

